# The prevalence of dental caries and its associated factors among preschool children in Huizhou, China: a cross-sectional study

**DOI:** 10.3389/froh.2024.1461959

**Published:** 2024-08-30

**Authors:** Jieyi Chen, Wanting Chen, Lude Lin, Haozhen Ma, Fang Huang

**Affiliations:** ^1^ Hospital of Stomatology, Sun Yat-sen University, Guangzhou, China; ^2^ Guangdong Provincial Key Laboratory of Stomatology, Guangzhou, China; ^3^ Guanghua School of Stomatology, Sun Yat-sen University, Guangzhou, China

**Keywords:** dental caries, epidemiology, oral health, tonsil, child

## Abstract

**Background:**

Dental caries among preschool children were prevalent worldwide and had a significant impact on children and their families. Understanding its prevalence and risk factors helps to optimize the delivery of oral health care to the target population and promote their oral health ultimately. This cross-sectional study aimed to examine the prevalence of dental caries and its associated factors among 3- to 5-year-old children in Huizhou, Guangdong Province, China.

**Method:**

We recruited children from 21 kindergartens adopting multistage sampling method. Two examiners performed oral examination. They assessed children's dental caries experience following the World Health Organization criteria. Children's dental caries activity, malocclusion, tonsil size and pH value of saliva were evaluated. Parental questionnaires collected child's sociodemographic background and oral-health-related behaviors. Data were analyzed by univariate analysis and logistic regression using SPSS.

**Results:**

This study invited 1,485 children and recruited 1,348 (53.2% boys) (response rate: 90.8%). Dental caries prevalence rate was 58.2% for 3-, 70.7% for 4-, 80.5% for 5-year-old and 72.9% for all recruited children. The mean dmft score (±SD) was 3.38 (±4.26) for 3-, 4.75 (±4.96) for 4-, 5.81 (±5.71) for 5-year-old and 4.99 (±5.02) for all children. Age, family status (singleton or not), monthly family income, mother and father's education level, tonsil grading score, spacing in dentition, Cariostat score (reflecting the caries activity), dental plaque index, duration of breastfeeding, dental visit experience, tooth brushing habits and sugary snacking before sleeping were statistically related to the prevalence of dental caries (*p* < 0.050) in univariate analysis. These factors were further analyzed in the regression model. The results of the final model indicated dental caries were associated with age (*p* < 0.001), Cariostat score (*p* < 0.001), spacing (*p* < 0.001), tonsil grading score (*p* = 0.013), singleton or not (*p* = 0.002), sugary snacking habit before bed (*p* < 0.001) and breast-feeding duration (*p* = 0.050).

**Conclusion:**

Dental caries was prevalent among 3-to 5-year-old preschool children in Huizhou, China. Children's age, caries activity, tonsil size, malocclusion, family background, sugary snacking habit and breast-feeding habit were related to the prevalence of dental caries. More emphasis should be placed on prevention targeting the risk factors from early life.

## 1 Introduction

Dental caries is the most prevalent chronic disease among children with a significant impact on individuals, families and society (1). Although it's largely preventable, more than 530 million children worldwide affected with dental caries in the primary dentition and most of the decayed teeth were untreated (2). The American Academy of Pediatric Dentistry defined “early childhood caries” (ECC) as the presence of one or more decayed (non-cavitated or cavitated lesions), missing (due to caries), or filled tooth surfaces in primary tooth of a child under 6 years old (3). A recent systematic review reported a global combined prevalence of ECC was 48% based on a variety of studies using WHO criteria for ECC prevalence (4). In China, the prevalence of ECC in children aged 3, 4 and 5 years is 50.8%, 63.6% and 71.9% respectively, reported by the latest National Oral Health Survey in China (5). The prevalence of dental caries among 5-year-olds was 5.8% higher than 10 years ago. Rural area had a higher prevalence rate when compared to urban area. ECC remained a serious concern for children in China (5). Untreated ECC not only critically affects the oral health of children, but also their general well-being. It may lead to localized pain, infection, early primary tooth loss, malocclusion, eating and speech disorders, as well as developmental delays (6–8). The early prevention of dental caries in preschool children is essential.

The occurrence and development of ECC is the result of a long-term imbalance of multiple risk and protective factors (7), such as sociological, biological, environmental factors and a wide range of oral health related behaviors. It is generally recognized that lower socio-economic status, poor parental education, lack of good oral hygiene practices and feedings of sugary foods at night are risk factors for caries (9–12). In addition, early life experience which affects children's growth and development may also linked to dental caries (13). One notable factor was breastfeeding. The relationship between breastfeeding practices and ECC has been recognized in recent studies but remains unclear and further research is required (14). The composition and properties of saliva also play a crucial role in the development and progression of dental caries. It may protect the teeth through various mechanisms, such as removal of food residues and sugars, aggregation and elimination of microorganisms, buffering by neutralizing acids, and antimicrobial defense. However, salivary dysfunction including low saliva flow rate and deficient buffering capacity would increase the incidence of dental caries (15). This study also assessed the relationship between palatine tonsils and dental caries. Palatine tonsils are located between the lingual and palatine arches, which are the largest peripheral immune organs in the oropharynx. Tonsillar hypertrophy is a common condition that occurs in 1%–3% of Chinese children aged 2–6 years (16), and its common etiology is currently thought to be prolonged inflammatory stimulation and closely related to the microbiota (17). Some studies have found that palatine tonsils hypertrophy leads to mouth breathing and causes orofacial problems in children. Mouth breathing might lead to a high risk of dental caries (18), but few studies reported the relationship between palatine tonsils and caries. Results of the present study may add new information of the relationship between palatine tonsils and dental caries.

China is a large country where has significant dietary and cultural differences between regions, which may affect children's oral health conditions. Besides, with China's rapid economic development, inequalities were found in children's health (19). Huizhou is a second-tier coastal city which located in the rapidly developing Pearl River Delta of Guangdong Province in southeastern China (20). The population of Huizhou is approximately 6 million, and about 80 percent are Hakka people (21) (a subgroup of the Han Chinese). Belonging to the Sino-Tibetan family, this ethnic group speaks the Hakka dialect and prefers Hakka cuisine, which is salty and aromatic. In 2021, Huizhou's Gross Domestic Product per capita was 70,191 yuan (about US$9,862), which was much lower than the national average (about US$12,551) (22). Until now, no reported epidemiological studies about the status of Huizhou preschool children's dental caries had been published. Moreover, there is no prevention strategies targeting dental caries for Huizhou children, such as topical fluoride application. This study was carried out to investigate the prevalence and associated factors of early childhood caries among 3- to 5-year-old preschool children in Huizhou, China. Results of this study may help to optimize the local delivery of individualized prevention and treatment, and to promote children's oral health in Huizhou.

## 2 Method

This is a cross-sectional study conducted from March to May 2022 following the Declaration of Helsinki, and reported in reference of the STrengthening the Reporting of OBservational studies in Epidemiology (STROBE) (Supplementary File 1) (23).

### 2.1 Ethics

Before the commencement of this study, ethical approval was obtained from the Medical Ethics Committee of the Hospital of Stomatology, Sun Yat-sen University (KQEC-2021-39-03), and the written parental consents of the participating children were obtained.

### 2.2 Subjects

The sample size was calculated using the formula: n=Z2×P×(1−P)/d2, where the *Z* statistic was confidence based, *P* was prevalence with d as precision (24). The fourth national oral health epidemiological survey in 2018 showed that the prevalence of dental caries in children aged 3, 4 and 5 years is 50.8%, 63.6% and 71.9% in China (25), respectively. With a confidence level of 95% and a confidence interval (CI) of 10% (40.5%–50.5%), giving an assumed response rate of 80%, a total of 1,320 children aged 3–5 years should be invited for this study.

To recruit children, multistage sampling method was adopted. All eight sub-districts in Huizhou were selected, and preschools in the sub-districts were categorized in to public and private kindergartens according to their regulatory bodies. In the first stage, the proportion of children invited in public and private kindergartens in each sub-district was determined by the proportion of public vs. private kindergartens and the proportion of the populations of the eight sub-districts distributed. In the second stage, a simple random sampling method was used to select public and private kindergartens in each district and used to select one class from each grade (K1, K2 and K3) in the selected kindergartens. Finally, all children in the selected classes were invited using the cluster sampling method. Children aged 3–5 years in all selected classes were recruited to enroll in the study based on the inclusion criteria: primary dentition, both parents are Han Chinese who are local residents in Huizhou, no medical history of serious systemic disease, no craniofacial abnormalities, and had good cooperation.

### 2.3 Questionnaire survey

A closed-ended parental questionnaire was designed based on the conceptual model affecting children's oral health that proposed by Fisher Owens and distributed (26). Parents or legal guardians completed questionnaires with no assistance and submitted before the day of the clinical examination of their children. The questionnaire consisted of the following four sections:

• Demographic background: age, sex, only-child or multi-child family, primary caregiver
• Socio-economic status: parental education level, family monthly income
• Oral health–related behaviours: dietary habits, toothbrushing habits, dental visit experience
• Child's feeding pattern: feeding type in the first 6 months of life, duration of breastfeeding and bottle-feeding, sleeping with a bottle

In the questionnaire, “occasionally” referred to 3 or less than 3 days a week while “often” referred to more than 3 days a week. An assistant checked the returned questionnaires and followed up on inappropriate and missing information in the questionnaires by telephone.

### 2.4 Oral examination

The dental examinations were carried out by two dentists [(LL and WC)] who were trained and calibrated by an experienced pediatric dentist (FH). Both examiners maintained excellent inter- and intra-examiner consistency, with a kappa value of over 0.9. A thorough examination of dental caries, plaque index, tonsil degree, crowding and spacing were carried out in kindergarten with the aid of a portable headlight, disposable mouth mirror and CPI probe with the child in the supine position. To assess excellent inter- and intra-examiner reliability, 5% of subjects were randomly selected for re-examination at the same day.

Dental caries was diagnosed following the World Health Organization (WHO) criteria. Children who had any decayed missing and filled teeth (dmft) were recorded as having caries and dmft score were calculated. The plaque index of six index teeth (55, 53, 51, 71, 73, 75) was assessed using the Silness and Löe scale (25). Tonsil size was classified according to the Friedman grading scale (27), with the following criteria: Zero was used to denote that the patient had a tonsillectomy. Grade I tonsils were in tonsillar fossa. Grade II tonsils were visible behind the anterior pillars. Grade III tonsils extended 3/4 of way to the midline, Grade IV tonsils were completely obstructing the airway. For crowding and spacing, if the overlap of the primary teeth is >2 mm, crowding is recorded, while if the wide interval between the primary teeth is >2 mm, spacing is diagnosed. No radiographs were taken during oral examinations.

### 2.5 Caries activity and salivary flow rate assessment

The Cariostat kit (GangDa Medical Technology Co. LTD., Beijing, China) was used to assess caries activities. According to the instruction, examiners scrubbed the buccal surfaces of the maxillary molars and mandibular incisors 3–5 times with a sterile cotton swab, and then placed the swab in a medium containing 2.5 ml of Cariostat test medium. Samples were incubated at 37°C for 48 h. The Cariostat score was assessed by comparing the reference colour provided by the manufacturer. The reference colour score is graded from 0 to 3, with intervals of 0.5 points. The colour changes from blue to green and eventually to yellow, indicating an ability to increase the acid production of plaque in the sample.

Then students were asked to rinse their mouths with water. Unstimulated saliva was collected by spitting the saliva through a funnel into a 5 ml tube within 10 min. The flow rate of unstimulated saliva was calculated (ml/min) accordingly. The buffering capacity of the saliva was measured by the Ericsson method (27). We added 0.5 ml of saliva to 1.5 ml of 5 mmol/L hydrochloric acid within 5 min, and left it stand for 20 min to remove carbon dioxide. The ultimate pH of saliva was assessed by using pH test strips. The buffering capacity of unstimulated saliva was recorded as “low”, “normal” or “high” when the pH was in the range of ≤3.5, 3.5–4.75 or ≥4.75, respectively.

### 2.6 Statistical analysis

The data were entered into personal computers by the examiners with Microsoft Office Excel 2013. Two field assistants double checked the data to avoid mis-entry. Missing data in questionnaires were filled by the median number. Data were analysed using SPSS version 25.0 for Windows (IBM Inc, Chicago, IL, USA). Frequency and percentage were presented. The intra-examiner agreement was evaluated using Kappa statistics. A Chi-square test was used to assess the differences in risk factors between the caries group and the caries-free group. The Mann-Whitney *U*-test or Kruskal–Wallis *H*-test was used to evaluate the distribution of dmft score under different variables because the dmft score did not follow a normal distribution. All risk factors with a *p*-value of less than 0.1 in the Chi-square test were included in the multivariate regression analysis to investigate the relationship between caries prevalence and the variables selected. The odds ratio (OR) and 95% CI were calculated. The statistical significance level for all tests was set at *p* < 0.05.

## 3 Results

A total of 1,485 children from 21 kindergartens were invited. Among them, 21 children did not have parental consent forms and were excluded. During the examination, 116 participants were uncooperative or absent. Finally, the study recruited 1,348 preschool children (response rate: 90.8%), among who, 717 were boys (53.2%). The mean age of these children was 4.26 [standard deviation (SD): 0.65] years.

### 3.1 Dental caries status

Results showed that the prevalence rate of dental caries was 73%, with a mean dmft score (±SD) of 4.99 ± 5.02. The untreated caries (dt = 4.85 ± 4.93) accounted for 97% of the dmft score, while the mean number of mt or ft accounted for a small percentage (mt = 0.01; ft = 0.13) (Table 1). The dental caries prevalence rates of the 3-, 4-, and 5-year-old children were 58.2%, 70.7%, and 80.5%, respectively. There was a statistically significant difference in the prevalence rate among the three age groups (*p* < 0.001). The mean dmft scores were 3.38 ± 4.26, 4.75 ± 4.96, and 5.81 ± 5.17 among 3-, 4-, and 5-year-old children, respectively. No differences in dental caries prevalence between sex were statistically significant (*p* > 0.05).

**Table 1:** Dental caries status of 3- to 5-year-old children in Huizhou, according to sex and age.

Independent variables (*n*)	Caries-free *N* (%)	Have dental caries *N* (%)	*p*-value^a^	Mean dt (±SD)	Mean mt (±SD)	Mean ft (±SD)	Mean dmft (±SD)	*p*-value
All children (1,348)	365 (27.0)	983 (73.0)		4.85 ± 4.93	0.01 ± 0.09	0.13 ± 0.67	4.99 ± 5.02	
Sex			0.22					0.358^b^
Boys (717)	184 (25.7)	533 (74.4)		4.96 ± 4.91	0.01 ± 0.09	0.11 ± 0.61	5.07 ± 5.02	
Girls (631)	181 (28.7)	450 (71.4)		4.73 ± 4.94	0.01 ± 0.08	0.15 ± 0.73	4.88 ± 5.03	
Age (year)			<0.001					<0.001^c^
3 (153)	64 (41.8)	89 (58.2)		3.30 ± 4.20	0.01 ± 0.08	0.07 ± 0.35	3.38 ± 4.26	
4 (697)	204 (29.3)	493 (70.7)		4.64 ± 4.89	0.01 ± 0.07	0.10 ± 0.63	4.75 ± 4.96	
5 (498)	97 (19.5)	401 (80.5)		5.61 ± 5.04	0.01 ± 0.10	0.19 ± 0.78	5.81 ± 5.17	

a Chi-square test; ^b^Mann–Whitney *U*-test; ^c^Kruskal–Wallis test.

**Figure 1:**
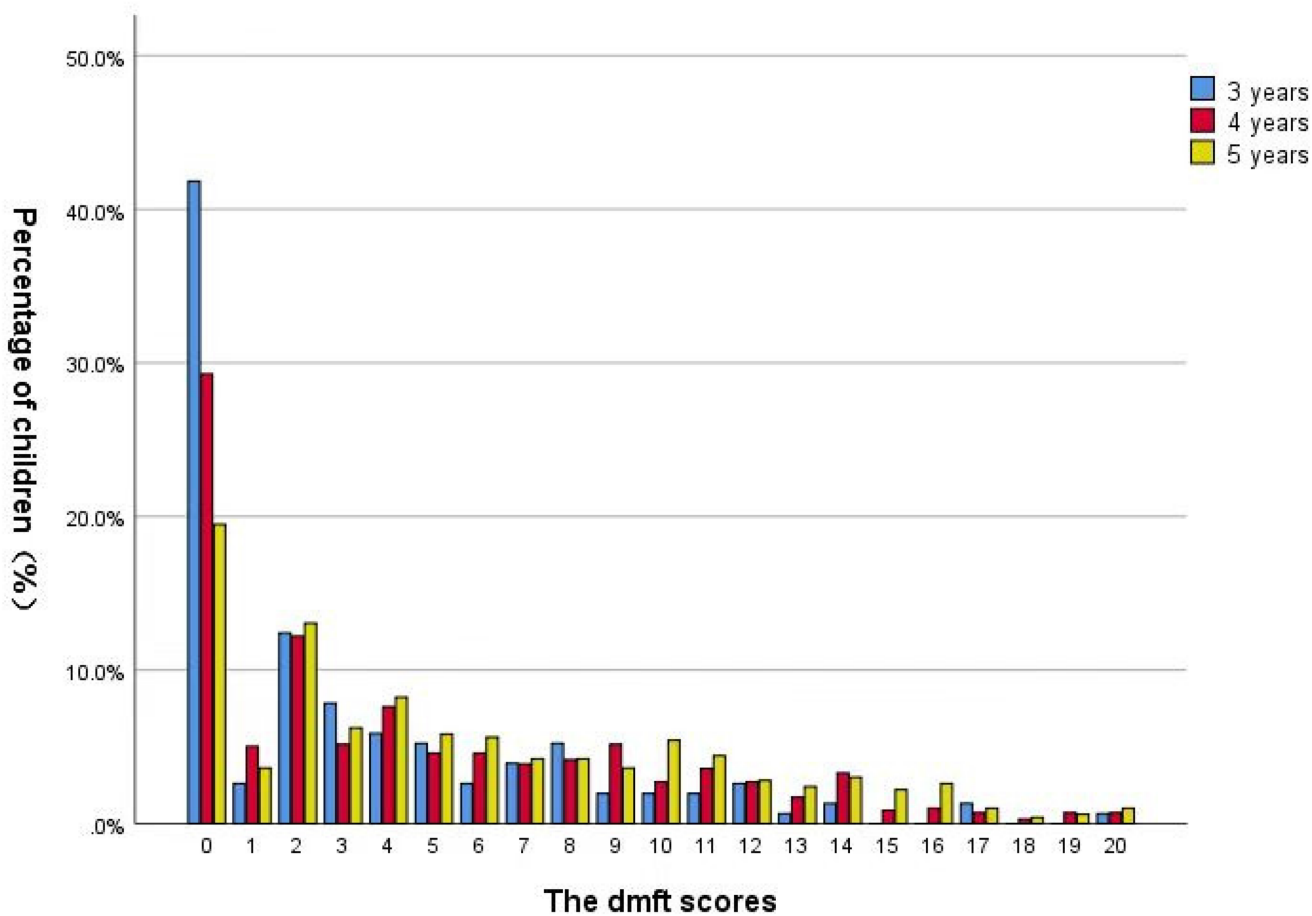
Distribution of dmft score among 3- to 5-year-old children in Huizhou, China.

**Figure 2:**
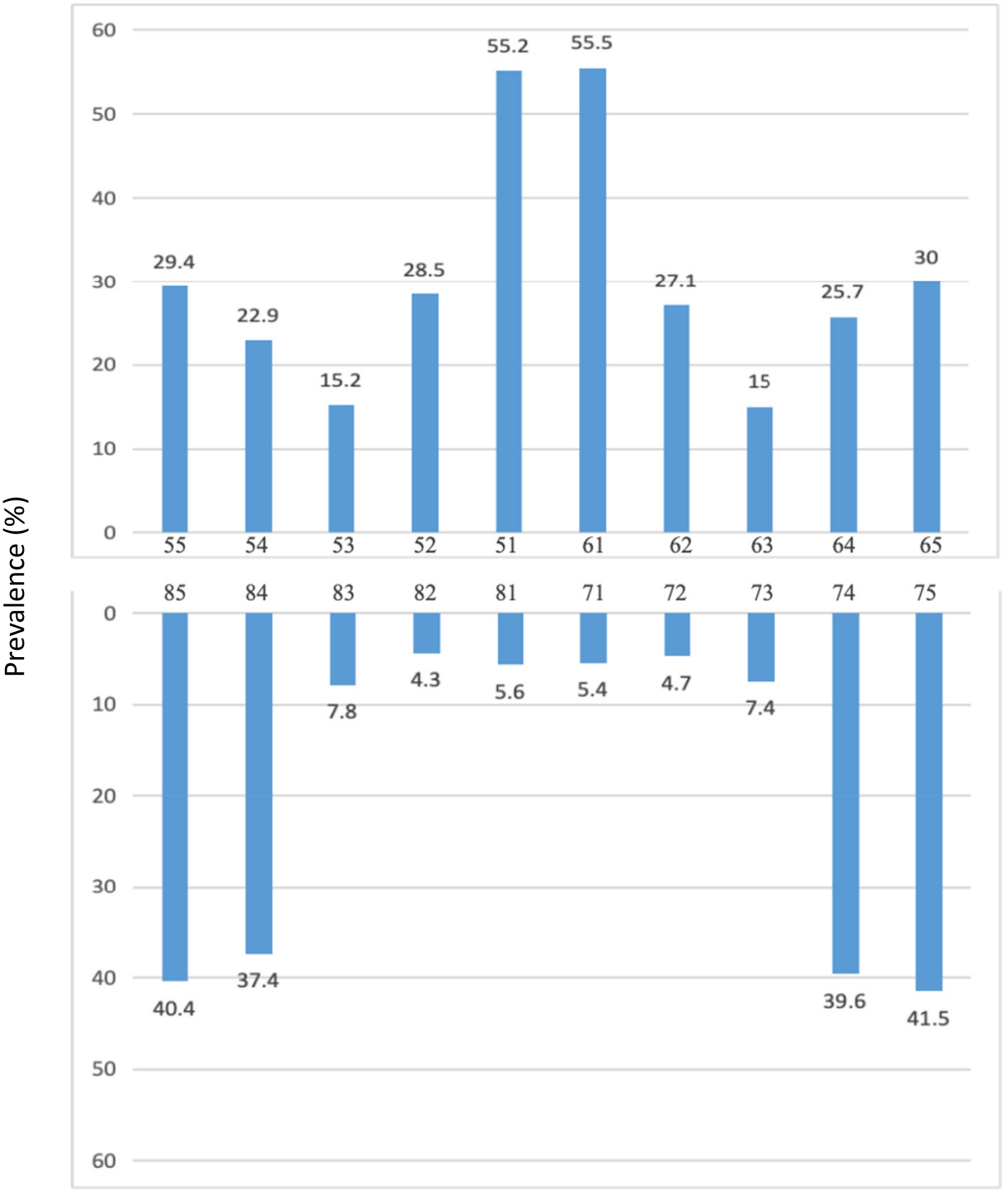
Distribution of teeth had dental caries according to tooth position.

The distribution of the dmft score was right skewed and shown in Figure 1. A majority of studied children had a dmft score of zero. Among children with dental caries experience, the mode dmft score was two. The number of participants tapered off slowly for higher dmft values. The distribution of dental caries in different tooth is shown in Figure 2. The dental caries prevalence rate of maxillary teeth was 68%, which was higher than that of mandibular teeth (58.2%). Maxillary central incisors had the highest prevalence rate of dental caries (55.5%), followed by mandibular molars (41.5%) and maxillary molars (30%), whereas mandibular lateral incisors had the lowest prevalence rate (4.3%).

### 3.2 Tonsil grading, caries activities and saliva assessment

Among the study children, 193 (14.3%) had a tonsil degree of III or above. Half children (54.3%) had spaces in the dentition, and 9.4% had crowding (Table 2). The majority (77%) of children had a salivary flow rate of ≥0.25 and approximately half (49. 7%) of the children within the normal range of unstimulated salivary buffering capacity. Besides, according to Cariostat score, 18.2% of the children had low acidogenic activity, while 32.8% had high acidogenic activity. Meanwhile, 14% of the children had a plaque index of 2 −3.

**Table 2:** The condition of tonsil grading, saliva physical properties, interdental spacing and crowding, plaque index and Cariostat score among children aged 3–5 years in Huizhou, China, according to age and sex [*N* (%)].

Independent variables (*n*)	Age (year)			*p*-value	Sex		*p*-value
	3	4	5		Male	Female	
Tonsil grading				0.943			0.043*
I (546)	58 (37.9)	285 (40.9)	203 (14.8)		271 (37.8)	275 (43.6)	
II (609)	74 (48.4)	313 (44.9)	222 (44.6)		331 (46.2)	278 (44.1)	
≥III (193)	21 (13.7)	99 (14.2)	73 (14.7)		115 (16.0)	78 (12.4)	
Spacing				0.433			0.001*
No (616)	77 (50.3)	318 (45.6)	221 (44.4)		298 (41.6)	318 (50.4)	
Yes (732)	76 (49.7)	379 (54.4)	277 (55.6)		419 (58.4)	313 (49.6)	
Crowding				0.310			0.344
No (1,220)	136 (88.9)	639 (91.7)	445 (89.4)		654 (91.2)	566 (89.7)	
Yes (128)	17 (11.1)	58 (8.30)	53 (10.6)		63 (8.80)	65 (10.3)	
Saliva secretion (ml/min)				0.000*			0.072
<0.25 (310)	54 (35.3)	162 (23.2)	94 (18.9)		151 (21.1)	159 (25.2)	
≥0.25 (1,038)	99 (64.7)	535 (76.8)	404 (81.1)		566 (78.9)	472 (74.8)	
Saliva buffering capability (pH)				0.000*			0.076
≤3.5 (354)	57 (37.3)	184 (26.4)	113 (22.7)		170 (23.7)	184 (29.2)	
3.5–4.75 (670)	74 (48.4)	354 (50.8)	242 (48.6)		369 (51.5)	301 (47.7)	
>4.75 (324)	22 (14.4)	159 (22.8)	143 (28.7)		178 (24.8)	146 (23.1)	
Plaque index				0.752			0.051
0–1 (444)	57 (37.3)	230 (33.0)	157 (31.5)		216 (30.1)	228 (36.1)	
1–2 (715)	76 (49.7)	367 (52.7)	272 (54.6)		392 (54.7)	323 (51.2)	
2–3 (189)	20 (13.1)	100 (14.3)	69 (13.9)		109 (15.2)	80 (12.7)	
Cariostat score				0.000*			0.208
0–1 (246)	37 (24.2)	143 (20.5)	66 (13.3)		122 (17.0)	124 (19.7)	
1–2 (659)	87 (56.9)	332 (47.6)	240 (48.2)		366 (51.1)	293 (46.4)	
2–3 (443)	29 (19.0)	222 (31.9)	192 (38.6)		229 (31.9)	214 (33.9)	

Chi-square test.

* *p* < 0.05**.**

### 3.3 Background information and oral health-related behaviours

More than half (55.1%) of the children never had a dental visit, and only 6.2% had regular dental visits. Most of the children (77.9%) had their mothers as their primary caregiver. A few (17.9%) children were from one-child families. Nearly half of the children (44.1%) consumed sugary snacks twice a day or above. Over half (63.3%) of the children started brushing their teeth at or before the age of two, and the majority (86.8%) brushed with parents’ assistance. A total of 31.1% children brushed their teeth at least twice a day, but only 23.5% of them did not eat sweets or sweetened beverages after teeth brushing. A few children (17.1%) adopted other cleaning aids such as flossing. Concerning the feeding pattern, the statistics show that the number of exclusively breastfed in the first 6 months was 51.5%, with artificial feeding at 13% and mixed feeding at 35.5%. Children who had breastfeeding lasted for more than 12 months accounted for 20.2%. Nearly half (45.6%) of the children were bottle-fed for more than 18 months. Only a few (5.2%) children had the habit of sleeping with bottle milk (Table 2).

### 3.4 Associated factors of dental caries

Results of the univariate analysis were presented in Table 3. No significant difference was found in the prevalence of dental caries or mean dmft score between boys and girls (*p* > 0.05). Factors that significantly associated with both dental caries prevalence and mean dmft score were age, dental plaque index, Cariostat score, tonsil degree, spacing, mother's educational level, one-child family, monthly household income, history of dental visits, time of starting brushing, daily frequency of brushing and sugary snacks before sleep (*p* < 0.05). Salivary buffering capacity, father's education level, frequency of daily snacks, duration of breastfeeding, sleeping with bottle milk and parents’ assistance in brushing were only associated with mean dmft score (*p* < 0.05).

Risk factors with a *p*-value of less than 0.10 in the Chi-square test were applied to a multivariate regression analyzed by the Forward LR method. Table 4 showed the relationship between selected variables and dental caries in the regression model. Results indicated that the prevalence of dental caries increased by age. Compared to the age of 3, the age of 4 and 5 had higher odds ratios, which were 1.644 (CI: 1.110–2.436) and 2.501 (CI: 1.638–3.819), respectively (*p* < 0.05). Besides, higher Cariostat score had a higher probability of having dental caries. Compared to a Cariostat score of 0–1, children who had Cariostat score of 2–3 had a 7-fold increased risk of caries (OR: 7.941, CI: 5.185–12.162, *p* < 0.001). In addition, children who had tonsil degree III and above were more likely to suffer from dental caries (OR = 1.874, CI: 1.218–2.881, *p* = 0.004). Regarding the variables of snacking habits, sugary snacks before sleeping was significantly associated with the prevalence of dental caries (OR = 1.789, CI: 1.331–2.405, *p* < 0.001 for occasionally, and OR = 1.778, CI: 1.037–3.047, *p* = 0.036 for often). As for feeding practices, children who were breastfed for more than 12 months were more likely to develop dental caries with an odds ratio of 1.711 (CI: 1.075–2.722, *p* = 0.024). In contrast, children with spacing in the dentition (OR = 0.572, CI: 0.437–0.748, *p* < 0.001), as well as from one-child family (OR = 0.597, CI: 0.432–0.824, *p* = 0.002), were less likely to have dental caries.

**Table 3:** Univariate analysis on factors related to dental caries among children aged 3–5 years in Huizhou, China.

Independent variables (*n*)	ECC-free (*n* = 365) *n* (%)	ECC (*n* = 983) *n* (%)	*p*-value^a^	Mean dmft (±SD)	*p*-value
Gestational age			0.950		0.803^c^
Preterm birth (106)	28 (26.4)	78 (73.6)		4.73 ± 4.99	
Term birth (1,154)	312 (27.0)	842 (73.0)		4.96 ± 4.95	
Post-term birth (88)	25 (28.4)	63 (71.6)		5.60 ± 5.86	
Saliva secretion (ml/min)			0.377		0.618^b^
<0.25 (310)	90 (29.0)	220 (71.0)		5.02 ± 5.32	
≥0.25 (1,038)	275 (26.5)	763 (73.5)		4.97 ± 4.93	
Saliva buffering capability (pH)			0.066		0.001^c^
≤3.5 (354)	96 (27.1)	258 (72.9)		4.75 ± 4.85	
3.5–4.75 (670)	166 (24.8)	504 (75.2)		5.48 ± 5.19	
>4.75 (324)	103 (31.8)	504 (68.2)		4.21 ± 4.72	
Plaque index			<0.001		<0.001^c^
0–1 (444)	154 (34.7)	290 (65.3)		3.51 ± 4.02	
1–2 (715)	175 (24.5) 36 (19.0)	540 (75.5)		5.27 ± 5.03	
2–3 (189)		153 (81.0)		7.35 ± 5.93	
Cariostat score			<0.001		<0.001^c^
0–1 (246)	115 (46.7)	131 (53.3)		2.32 ± 3.16	
1–2 (659)	211 (32.0)	448 (68.0)		4.05 ± 4.59	
2–3 (443)	39 (8.8)	404 (91.2)		7.86 ± 5.13	
Tonsil grading			0.026		0.04^c^
I (546)	158 (28.9)	388 (71.1)		4.65 ± 5.81	
II (609)	170 (27.9)	439 (72.1)		5.11 ± 5.23	
≥III (193)	37 (19.2)	156 (80.8)		5.53 ± 4.86	
Crowding			0.792		0.628^b^
No (1,220)	332 (27.2%)	888 (72.8%)		5.01 ± 5.03	
Yes (128)	33 (25.8%)	95 (74.2%)		4.70 ± 4.89	
Spacing			<0.001		<0.001^b^
No (616)	136 (22.1%)	480 (77.9%)		5.87 ± 5.41	
Yes (732)	229 (31.3%)	503 (68.7%)		4.24 ± 4.54	
Mother's education level			0.008		0.002^c^
Junior or below (297)	60 (20.2)	237 (79.8)		5.70 ± 5.26	
High or secondary (468)	130 (27.8)	338 (72.2)		5.10 ± 5.00	
Tertiary or above (583)	175 (30.0)	408 (70.0)		4.53 ± 4.87	
Father's education level			0.063		0.004^c^
Junior or below (261)	64 (24.5)	197 (75.5)		5.72 ± 5.47	
High or secondary (489)	120 (24.5)	369 (75.5)		5.18 ± 4.97	
Tertiary or above (598)	181 (30.3)	417 (69.7)		4.50 ± 4.81	
Primary caregiver			0.711		0.249^c^
Father (41)	12 (29.3)	29 (70.7)		4.05 ± 4.39	
Mother (1,051)	279 (26.5)	772 (73.5)		5.08 ± 5.01	
Other people (256)	74 (28.9)	182 (71.1)		4.74 ± 5.16	
One-child family			0.001		<0.001^b^
No (1,106)	275 (24.9)	831 (75.1)		5.18 ± 5.05	
Yes (242)	90 (37.2)	152 (62.8)		4.10 ± 4.79	
Monthly family income (RMB)			0.018		0.051^c^
≤4,000 (141)	33 (23.4)	108 (76.6)		5.55 ± 5.31	
4,001–8,000 (317)	67 (21.1)	250 (78.9)		5.41 ± 5.11	
8,001–12,000 (471)	143 (30.4)	328 (69.6)		4.80 ± 4.92	
>12,000 (419)	122 (29.1)	297 (70.9)		4.68 ± 4.95	
Feeding methods (first 6 months)			0.185		0.443^c^
Exclusive breastfeeding (695)	175 (25.2)	520 (74.8)		5.14 ± 5.09	
Exclusive bottle-feeding (174)	55 (31.6)	119 (68.4)		4.70 ± 4.83	
Mixed feeding (479)	135 (28.2)	344 (71.8)		4.87 ± 4.99	
Duration of breastfeeding			0.055		0.038^c^
<1 month (175)	56 (32.0)	119 (58.0)		4.67 ± 4.83	
1–6 months (360)	109 (30.3)	251 (69.7)		4.46 ± 4.81	
6.1–12 months (540)	139 (25.7)	401 (74.3)		5.20 ± 5.15	
>12 months (273)	61 (22.3)	212 (77.7)		5.44 ± 5.11	
Duration of bottle-feeding			0.503		0.706^c^
0–12 month (381)	99 (26.0)	282 (74.0)		5.07 ± 4.94	
12.1–18 months (352)	90 (25.6)	262 (74.4)		5.00 ± 4.96	
>18 months (615)	176 (28.6)	439 (71.4)		4.93 ± 5.11	
Sleeping with bottle (milk or sweetened drink in bottle)			0.376		0.001^b^
No (1,277)	349 (27.3)	928 (72.7)		4.84 ± 4.89	
Yes (71)	16 (22.5)	55 (77.5)		7.51 ± 6.44	
Frequency of dental visit			0.022		<0.001^c^
Never (744)	220 (29.6)	524 (70.4)		4.50 ± 4.82	
Irregularly (520)	119 (22.9)	401 (77.1)		5.80 ± 5.28	
Regularly (84)	26 (31.0)	58 (69.0)		4.21 ± 4.35	
Age of first dental visit			0.028		<0.001^c^
Never (744)	220 (29.6)	524 (70.4)		4.50 ± 4.82	
<3 years (331)	87 (26.3)	244 (73.7)		5.01 ± 4.91	
≥3 years (273)	58 (21.2)	215 (78.8)		6.27 ± 5.45	
Age that starts tooth brushing			0.001		0.001^c^
6 months (93)	40 (43.0)	53 (57.0)		3.26 ± 4.19	
1-year-old (227)	65 (28.6)	162 (71.4)		4.94 ± 5.21	
2-year-old (533)	144 (27.0)	389 (73.0)		4.96 ± 5.04	
3-year-old and above (495)	116 (23.4)	379 (76.6)		5.35 ± 5.00	
Brushing frequency daily			0.011		0.051^c^
<1 on average or never (248)	65 (26.2)	183 (73.8)		5.52 ± 5.22	
1 (680)	164 (24.1)	516 (75.9)		4.96 ± 4.80	
≥2 (420)	136 (32.4)	284 (67.6)		4.71 ± 5.23	
Parents’ assist on brushing			0.153		0.013^c^
No (179)	45 (25.1)	134 (74.9)		4.64 ± 4.63	
Occasionally (828)	214 (25.8)	614 (74.2)		5.30 ± 5.13	
Often (342)	106 (31.1)	235 (68.9)		4.40 ± 4.87	
Use of dental floss or mouthwashes			0.564		0.961^b^
No (1,117)	306 (27.4)	811 (72.6)		5.02 ± 5.08	
Yes (231)	59 (25.5)	172 (74.5)		4.83 ± 4.74	
Sugary snacks before bed			<0.001		<0.001^c^
Never (318)	120 (37.7)	198 (62.3)		3.72 ± 4.48	
Occasionally (926)	218 (23.5)	708 (76.5)		5.34 ± 5.09	
Often (104)	27 (26.0)	77 (74.0)		5.66 ± 5.31	
Frequency of snacking daily			0.317		0.005^b^
2 times or less (753)	212 (28.2)	541 (71.8)		4.62 ± 4.85	
More than2 times (595)	153 (25.7)	442 (74.3)		5.45 ± 5.20	

a Chi-square test; bMann–Whitney *U*-test; cKruskal–Wallis test.

**Table 4:** Logistic regression of variables related to dental caries among children aged 3–5 years in Huizhou, China.

Independent variables (*n*)	OR	95%CI		*p*-value
		Lower	Upper	
Age (year)				<0.001*
3	Ref			
4	1.644	1.110	2.436	0.013*
5	2.501	1.638	3.819	<0.001*
Cariostat score				<0.001*
0–1	Ref			
1–2	1.675	1.224	2.293	0.001*
2–3	7.941	5.185	12.162	<0.001*
Spacing				<0.001*
No	Ref			
Yes	0.572	0.437	0.748	
Tonsil grading				0.013*
I	Ref			
II	1.036	0.783	1.371	0.806
≥III	1.874	1.218	2.881	0.004*
One-child family				0.002*
No	Ref			
Yes	0.597	0.432	0.824	
Sugary snacks before sleeping				<0.001*
Never	Ref			
Occasionally	1.789	1.331	2.405	<0.001*
Often	1.778	1.037	3.047	0.036*
Duration of breastfeeding				0.050*
<1 month	Ref			
1–6 months	1.070	0.698	1.638	0.757
6.1–12 months	1.387	0.925	2.078	0.113
>12 months	1.711	1.075	2.722	0.024*

* *p* < 0.05.

## 4 Discussion

This cross-sectional study is the first epidemiological study to investigate the dental caries status among preschool children in Huizhou, China. This study found the prevalence rate of dental caries and mean dmft score among the study population remained high. A significant gap existed between the prevalence rate of dental caries among Huizhou preschooler and the goal set by the WHO in 2003 which was that the caries-free rate of 5-year-old children would be 50% by 2020 (28). Moreover, differences in dental caries prevalence rate could be found between the study population and their peers in other countries such as India (50%) (29), Italy (19%) (30), South Africa (45%) (31) and United States (19%) (32). Though several dental caries management strategies were proposed and implemented in China (33), children in Huizhou, a second-tier city in Guangdong Province, seemed not benefit a lot from the current policies. The prevalence of dental caries of the study children was slightly higher than the national average which was reported in 2015 (prevalence: 71.9%, mean dmft: 4.24). It was also higher than other cities/provinces around China such as Shanghai (47.02%) (34), Hong Kong SAR (46%), Sichuan (63.47%) (35) and Guizhou (63.1%) (36), but similar to Zhejiang (70.4%) (37) and Yunnan (74.3%) (38). Besides, it was also higher than the average of Guangdong Province (68.3%) which was reported in 2016, and another northern city in Guangdong (3 year: 39.2%, 4 year: 44.8%) (39). The main results indicated that dental caries status among Huizhou preschool children was not optimistic and concerns from related stakeholders should be raised to improve the situation.

To manage dental caries, strategies that targeting risk factors should be taken into consideration. This study found several oral health-related behaviours were associated with dental caries. Sugary snacks before sleeping is a risk factor that commonly reported (30, 40, 41). The results showed participants who consumed sweets at bedtime were 1.78 times more likely to develop dental caries than those who did not. Sugary snacks before sleeping provides an large amounts of substrate (sucrose, glucose, and fructose) and sufficient time for the bacteria to produce acidic products, thus accelerates the development of dental caries (42). Similarly, breastfeeding also related to the development of dental caries. Our study found children who were breastfed for a long period of time were at a higher risk of developing dental caries, which was similar to other studies conducted in China (37, 43). Another study in Brazil reported that children who breastfed beyond 24 months had a 2.4-fold increased risk of severe dental caries when compared to children who breastfed for 1 year (44). Therefore, restricting sugary food before sleeping and the duration of breastfeeding may help to improve the situation of dental caries among preschool children. Though the WHO encourages exclusive breastfeeding for the first 6 months and complementary breastfeeding for the first 2 years (45), children's teeth should be cleaned and the frequency of sugary foods should be reduced (46) The British Society of Pediatric Dentistry even published restrictions on breastfeeding beyond 12 months in 2018 to avoid dental caries (limiting night feedings, etc.) (47). Oral health education which targeting the feeding before bed should be considered to provide to caregivers.

At the meantime, effective home tooth cleaning techniques should also be integrating into oral health education for Huizhou children as well as their caregivers. This study found that the plaque index and parent's assist in tooth brushing were related to the prevalence of dental caries in the univariate analysis but adjusted in the multivariate analysis. However, Cariostat score was statistically significant related to dental caries in the final model. Cariostat was designed to measure the pH value decrease caused by microorganisms in the plaque sample to assess dental caries activity (48). Children with higher Cariostate score would have higher level of cariogenic bacteria and have a higher chance to develop dental caries (49). Thus, effective plaque removal is essential to reduce the risk of dental caries among young children. Besides, the use of dental floss in young children should also be taken into consideration. Our study evaluated the relationship between interdental spacing and crowding with dental caries and found that interdental spacing was negatively correlated with caries, similar to the results reported by Cho (50). The absence of interdental spaces may complicate the cleaning of contact areas for young children and their parents, and children with spacing in their dentition may easily clean these areas and had less chance to develop dental caries on proximal surfaces. Though the relationship between crowding and dental caries was not found due to the small number of crowding of the study sample (9.4%), flossing should also be considered for caries prevention in children without interdental spacing.

Other social and biological factors were also found to be related to dental caries including age, number of children in the family, and tonsil grading in the final model. Attention should be paid to, and intervention strategies should be adopted for children with these factors. Previous studies reported similar findings. For example, elder children had higher chance to have higher prevalence and more severity of dental caries, consistent with other investigations (37). Caries is an irreversible process, and if cariogenic factors remain in affected children, this cumulative effect may contribute to the development of caries over time. This suggested that the prevention of dental caries should began at an early age. Prevention in childhood, in addition to being synonymous with monitoring the oral health of the child, means first of all to pay attention to parents who are the main behavioral reference (51, 52). A systematic analysis of studies has shown that people with lower parental educational backgrounds or incomes are more likely to had dental caries experience (53). Well-educated parents are likely to have a high level of dental knowledge, and probably provide more supervision and guidance during their daily oral health practice (54). Therefore, it's necessary to launch a parental oral health promoting program to control the risk factor of dental caries among children (55). Though this study found no significant relationship between family economic status, parental education, primary caregiver and dental caries in the final model, children who were from one-child families had lower chance to have dental caries. The reason could be that parents had only one child would have more time and pay more attention to their children's oral condition.

One interesting finding of this study was that 14.3% of children had enlarged palatine tonsils, and tonsil degree III and above increase the risk of dental caries. The palatine tonsils are a pair of flat oval lymphoid organs located between the lingual and pharyngeal palatine arches, which are adjacent to the oral cavity. The enlarged palatine tonsils lead to pharyngeal obstruction, causing problems such as snoring, dysphagia, mouth breathing and orofacial problems (56). A study of Chinese 3- to 6-year-old children found that the surface of the tonsils was richest in microbiota and shared with the oral cavity, regardless of tonsil health or under hypertrophic conditions. But no significant differences were found in the dmft index and dmfs index in the group with hypertrophic tonsils compared to normal tonsils (57). While Ahmed reported that the mean dental caries values were higher in children with chronic tonsillitis compared to those with normal tonsils, which may be due to the fact that caries and peritonsillar infections share the same microbial pathogens, such as Streptococcus mutans (58). In addition, tonsillar hypertrophy can be accompanied by mouth breathing, which leads to saliva reduction through evaporation. The reduction in resting saliva flow leads to an increase in the number and species of Candida and Lactobacillus bacteria, raising the risk of dental caries (59). More longitudinal clinical studies and experimental studies are needed to confirm the causal relationship between tonsil hypertrophy and dental caries. Moreover, this study also found sex were significant related to tonsil grading in the univariate analysis, which is in accord with other publications that more females present with tonsillitis compared to males and the reason is still under debate (60). Studies that aim at investigating the causal relationship between tonsil grading and sex may be needed in the future.

This study had other findings which may raise different stakeholders’ concerns, though these factors were not statistically significant related to dental caries in the analysis. One important factor was the utilization of dental services. Huizhou government requires that its local hospitals have to conduct annual oral examinations of preschool children in kindergartens, yet the study results showing that only half (55.1%) of the children had a dental visit, almost all of the caries (97%) were untreated. This indicates low utilization of dental services, and preventive techniques such as topical fluoride application and fissure sealing have not been widely promoted among preschool children in Huizhou. Moreover, the frequency and location distribution of dmft showed that the mode of dmft score was two, with the upper mesial incisors having the highest incidence, followed by posterior teeth, and mandibular lateral incisors had the lowest. Dental caries in anterior deciduous teeth may be associated with eruption at an early age, poor feeding habits and fail to take proper oral hygiene practice, whereas dental caries on molar teeth may be associated with deeply fissured and fossaed occlusal surfaces or compacted interdental space, which are difficult to clear. Therefore, promoting the utilization of dental service to provide preventive and therapeutic measures such as topical fluoride application annually, timely pits and fissure sealant may be essential to reduce the burden of dental caries among preschool children in Huizhou, China.

Overall, this study successfully enrolled a representative sample with a sufficiently large sample size and a high response through multistage sampling. Besides, this study followed WHO recommendations to maintain the representativeness, reliability and validity. Two examiners completed adequate calibration training before the start of this study and were obtained good internal and intra-examiner agreements. One limitation of this research is that the questionnaires may had potential recall bias from parents. In this study, caries was diagnosed according to WHO criteria rather than the International Caries Detection and Assessment System (ICDAS) so that the results can be compared to other studies. Radiographs were not used in the kindergarten setting, which may contribute to under estimation of caries status. It is worth noting that the diagnosis of interdental spaces may be influenced by adjacent caries, leading to an overestimation of interdental spaces. Another major limitation is that due to the nature of cross-sectional studies, our results can only find the association between risk factors and dental caries, but not the causative relationship between them. Further studies are needed to dynamically investigate the relationship between its influencing factors and dental caries. Nevertheless, this study is the first comprehensive analysis of dental caries among preschool children in Huizhou. The results may help epidemiologists and clinicians to better understand the prevalence and associated factors of dental caries among preschool children in Huizhou, China.

## 5 Conclusion

In conclusion, the prevalence of dental caries among children aged 3–5 years in Huizhou, China was high, and above the national average. Most of cavities remained unrestored. Children's age, caries activity, tonsil size, spacing, family background, sugary snacking habit and breast-feeding habit were related to the prevalence of dental caries. Emphasis should be placed on dental caries prevention from early life.

## Data Availability

The raw data supporting the conclusions of this article will be made available by the authors, without undue reservation.
